# Patient-Related Barriers to the Prescription of Cannabinoid-Based Medicines in Palliative Care: A Qualitative Approach

**DOI:** 10.1089/pmr.2022.0021

**Published:** 2022-09-16

**Authors:** Pauline Kalonji, Aurélie Revol, Barbara Broers, Michael Ljuslin, Sophie Pautex

**Affiliations:** ^1^ Department of Readaptation and Geriatrics, University of Geneva, Geneva, Switzerland.; ^2^ Palliative Medicine Division and University Hospital Geneva, Geneva, Switzerland.; ^3^ Primary Care Division, University Hospital Geneva, Geneva, Switzerland.

**Keywords:** cannabinoids, medical cannabis, palliative care, patients', expectations, patients', experiences, patients', perceptions

## Abstract

**Background:**

A minority of palliative care patients benefit from prescribed cannabinoid-based medicines (CBMs).

**Objective:**

The objective of this study was to explore the perceptions, expectations, and experiences of CBM usage among palliative care patients and to evaluate whether and how they may constitute an obstacle to prescription.

**Design:**

This is a qualitative study involving semistructured in-depth interviews with 10 patients hospitalized in a palliative care unit in Geneva, Switzerland. The data were analyzed using the interpretative phenomenological analysis method.

**Results:**

Semistructured interviews were conducted on 10 patients (average age of 73.3 years), mainly with advanced cancer. Most patients favored CBM use in palliative care and distinguished it from recreational use. Seven themes were identified from patients' perceptions, experiences, and expectations during the interviews: right time to begin CBMs, off-label use, information about side effects, lack of a safe medical framework, costs, relatives, and social acceptance of CBMs.

**Conclusion:**

The obstacles described by the patients seem to be surmountable with specific measures at the clinical level. We suggest training health professionals in a palliative care setting, especially in explaining the effects and side effects. CBMs will undoubtedly play a more significant role in palliative care medicine in the years to come.

## Introduction

Patients with advanced disease may have a significant symptoms burden that causes distress.^1^ A wide range of medications are available, but managing symptoms such as pain, nausea, anorexia, and anxiety remains an ongoing challenge for many palliative care patients.^2^

There is an increasing interest in the use of medicinal cannabinoids for the relief of symptoms in palliative care by patients' clinicians and strong public pressure.^3^ Cannabinoid-based medicines (CBMs) could potentially affect pain, sleep disorders, tiredness, anxiety, depression, anorexia, and nausea–vomiting induced by chemotherapy. Moreover, they could help relieve the end-of-life's emotional, existential, and spiritual suffering.^4^ However, prescription of cannabinoids is limited due to a lack of quality and quantity of evidence,^5–11^ and palliative care health professionals tend to underprescribe CBMs, even though they recognize that the treatment could be helpful.^12–15^

Moreover, some administrative burdens exist. For example, in Switzerland, currently, there is only one registered natural cannabis medication, nabiximols, an oral spray to relieve treatment-resistant spasticity associated with multiple sclerosis.^16^ For other medical indications and galenic forms of CBMs (oils, tinctures), the responsible physician must apply for a special authorization for each patient to the Federal Office of Public Health (FOPH). In addition, the costs of the (expensive) treatment remain the responsibility of the patients.^17^

In addition, few studies address how patients' and their families' views and representations of medical cannabis may influence its usage in palliative care.^18–20^ Therefore, the objective of this study was to explore the perceptions, expectations, and experiences of CBM usage among palliative care patients and to evaluate whether and how they may constitute an obstacle to prescription in a palliative care setting.

## Methods

### Design

This is a qualitative study that entails face-to-face semistructured interviews conducted between September 2020 and June 2021 in two palliative medicine units of the University Hospital Geneva (HUG), comprising 28 beds.

### Participants

Inclusion criteria were that participants be >18 years of age, fluent French speaking, and hospitalized in a palliative care unit in HUG. Exclusion criteria were acute delirium and the last days of life. Patients were randomly proposed by the team in charge of the units. Seven patients declined after being identified by the team in charge. The inclusion of patients was stopped when the researchers (S.P., A.R.) found that they had enough data to develop an understanding of the research question. There were no inclusion criteria regarding the recreational use of cannabinoids or the prescription of medical cannabinoids.

### Procedure

A medical student with no clinical relation to patients approached the participants, explained the study, obtained their written consent, and included them. She then conducted individual face-to-face interviews. She was supervised throughout the entire project by two researchers experienced in qualitative research (S.P., A.R.). We organized at least one meeting per week. The semistructured interview guide covered patients' perceptions of cannabinoids, experiences, and expectations or fears about CBMs (Appendix 1).

The interview guide was created in four steps: first, the medical student (P.K.) did a literature review to identify the main important topics and made the first draft. Second, the authors agreed on the last version of the interview guide. Third, the interview was tested with two patients and adapted according to their suggestions. Finally, interviews were audio-recorded, transcribed, and analyzed using the Nvivo software.

### Data analysis

All identifiable information was removed from the transcripts, and the qualitative analysis was conducted using the interpretative phenomenological analysis method.^17^ This method relies on discourses to understand the individual experience and its meaning. It allows revealing the intra- and interindividual elements and the social factors that influence behavior. The study professionals, unrelated to the patient's care, identified trends between patients' discourses: experience and knowledge of CBMs; alternatives to conventional medicines; addiction; prescription; and family, social, and societal environment. The analysis was performed independently by three members of the research team (P.K., S.P., A.R.), who then met regularly to discuss the coding structure.

The first step was for the authors to familiarize themselves with the data: listening to recorded interviews and reading and rereading transcripts to obtain an overall impression and become familiar with the text. The second step was the identification of subcategories and the designation of initial codes. In the third step, the codes were sorted into different themes. According to the fourth step, all the preliminary subcategories and themes were put together, compared, and grouped into categories. The fifth step of the analysis process included the discussion of subcategories and themes until consensus was reached, and each theme had a clear definition and a name.

Finally, narratives were extracted from the data material to represent categories and subcategories. The analysis was the object of regular meetings of the coauthors to revise and agree on the findings before going further in the thematic analysis and approval of results. Data collection and analysis were conducted simultaneously until no further news items emerged to reach the theoretical saturation, defined as when no new information was collected, no new themes could be detected, and responses tended to repeat. The data codes and themes were constantly checked, compared, and contrasted. Interdisciplinary team meetings were held to discuss core categories and themes.

### Ethics approval and consent to participate

The Geneva cantonal research committee approved the protocol. Patients received written and verbal information and signed the consent for participation in the research. The team in charge of the patients was able to support the patient after the interview if needed. All interviews were anonymized directly during the transcription.

## Results

### Participants

The final sample consisted of 10 patients, 4 women and 6 men, with an average age of 73.3 years (55–97). Eight patients had an advanced stage of cancer, and two had severe pulmonary diseases. Four patients were recreational users, two were on regular use, and two were occasional users. No patients had a prescription for CBMs at the time of the interviews.

### Themes

Seven themes were identified from the patient's experiences, representations, and expectations during the interviews: (1) right time to begin CBMs, (2) off-label use, (3) information about side effects, (4) lack of a safe medical framework, (5) costs, (6) relatives, and (7) social acceptance.

#### Right time to begin CBMs

The decision of when CBMs should be prescribed is undoubtedly delicate and divided the participants in the study.

Six patients were, in theory, quite reluctant to introduce CBMs. However, with sufficient information and without other therapeutic options, they could agree to use it to alleviate physical or psychological distress.

I'm against [medical cannabis] because it's a drug. […] If they have to use it … yes. I don't want to criticize them. No judgment […] If the person needs it, he needs it, but if not… (Patient 5)

Four patients were favorable but wanted the treatment to be introduced only when necessary. These patients were not interested in adding a product when they had no particular complaints or were satisfied with their current therapies.

At the moment, I don't feel the need for it. But if I start to have pain even when I'm in bed, that would change things […] The only situation for me to take it is to have pain even when I'm not being touched or moved. If medical cannabis could help me, I wouldn't hesitate. (Patient 8)

Finally, recreational cannabis smokers opposed cannabinoid treatment, doubting its effectiveness in relieving the severe pain experienced during their illness. They attributed the medical cannabis product to being less effective at fighting pain than the products they usually consume since recreational cannabis has a much higher content of delta-9-tetrahydrocannabinol (THC), the psychoactive substance in cannabis.

I have heard that the grass we find in the hospital has a very low THC percentage and that you have to take astronomical amounts to have a minimum effect, while on the street, it is stronger. It would be better to have something from the street to be effective. Or to change what they offer in the hospital or increase the doses. (Patient 3)

Furthermore, one former consumer reported a broader spectrum of effects, ranging from psychic—relaxation, letting go, cheerfulness, and decreased anxiety—to somatic impacts—increased appetite. Therefore, the right time for the introduction might depend on these factors.

It makes me see life more lightly, like a meditative state. You are not in the permanent thoughts of your small or big problems. You can more easily relax because you are in a situation where you are good. Or you forget that you are sick, that you are going to die, that you have pain […] It opens my appetite also. Now, I do not eat because I am very nauseous, but smoking cannabis makes me feel hungry. (Patient 1)

#### Off label use

Eight patients knew that cannabis is used as an off-label drug for its analgesic, anxiolytic, antidepressant, antiemetic, orexigenic, and hypnotic effects. Their expectations were clear, high, and far exceeded the primary effects of recreational cannabis.

Patients shared the hope that medical cannabis could be registered a medication to offer an alternative, particularly to psychotropic drugs. This point was recurrent in patients who were reluctant to accept the synthetic pharmacopeia and would prefer more natural options. Moreover, a recognized medical cannabis formulation would minimize the adverse effects of the route of administration. For example, drops or tablets let patients avoid smoking cannabis, affecting how well the lungs work.

Smoking a medication is out of the question. I have a severe COPD, so anything that directly affects my lungs is excluded. (Patient 8)

Seven patients have expectations concerning CBMs. Still, four believe it should be limited to situations of other treatment failures, cannabis referring to illegality, delinquency, and addiction.

#### Information about side effects

Eight patients consider that cannabis does not cause physical dependence or withdrawal syndrome, unlike “hard drugs.” But, according to them, individual predispositions would make them susceptible to increasingly potent drugs.

However, consumers of recreational cannabis list side effects: psychomotor slowdown, drowsiness, a feeling of discomfort, anxiety, and rarely “bad trip.”

Overall, patients feel uninformed about the adverse effects of cannabinoids.

The reactions are not very well known. I bought some drops, but I never used them. I did not dare. I didn't know the side effects. (Patient 10)

#### Lack of safe medical framework

The main concern was the product quality guarantee with stringent controls, clear medical indications, adequate dosage monitoring by physicians, and prescribed in a safe medical framework.

As part of a medical prescription, I am not against it. In a medical setting, controlled, yes. If I buy it around the corner, it's not okay, and it fuels delinquency. But in a setting like here at the hospital with a program, something planned, yes. (Patient 9)

#### Costs

Beyond the health aspects, patients fear that a CBM generates too high a cost as it is not covered by health insurance. In addition, recreational users consider that the medical cannabis product is much more expensive than the product they can obtain on parallel markets.

My fear is that it will not be reimbursed. Because I don't take high dosage and I'm limited financially. (Patient 1)

#### Relatives

The factors influencing the acceptance of CBMs are not only individual but are caught in a web of representations conveyed by the family, social and political environment. For example, if a CBM was a medical prescription, patients think their relatives would respect their choice.

My family and those around me did not like it, but now that I have something serious, I don't think they would say anything. They understood the value it could have. I smoked, and sometimes it was better. (Patient 3)

#### Acceptance of CBMs

On a sociological level, three patients feel that generational and socioeconomic factors mainly determine acceptance of CBMs. Younger patients, between 55 and 66 years, with previous personal experience with cannabis, are more open to more extensive scale development. In addition, three patients are convinced that political decisions influence public opinion about CBMs.

People who took cannabis were difficult. Those who wanted to take drugs, they took drugs, but it was always done secretly, they were condemned. It was forbidden. (Patient 6)

To remove these obstacles to access to CBMs, patients agree that the legalization of cannabis could provide a solution.

I think it should have been legalized for a long time, and today it's a witch hunt for nothing. There are many states in the world that have legalized the use of these products. (Patient 5)

## Discussion

Our qualitative interviews with 10 patients hospitalized in a palliative care setting suggest that most of them would accept using CBMs. By chance, four patients were recreational users of cannabis. However, they considered a prescription for CBMs unnecessary during the interviews. This might reflect the recommendations of prescription of CBMs to palliative care patients as a third-line treatment and not as a first- or second-line treatment and the lack of criteria on when to initiate the therapy.^18^

The attitude of recreational cannabis users could also be related to the difference in the administration form and the fact that they think the oral form is less effective.^21^ Still, patients seem to want to be informed at an early stage of their care, including discussing the potential benefits, side effects, and depending on the individuals' previous experiences with cannabinoids.^10,22^

About the timing of the introduction, the included patients were unfamiliar with the medical use of cannabinoids. They relied on their representations or experiences of nonmedical use of cannabis to justify prescription or nonprescription. Patients reported a lack of medical information on cannabinoids, both theoretical and clinical. Questions about the monitoring and the routes of administration were frequent. In a survey of palliative care providers, Luba showed a similar ambivalence.^13^

Caregivers endorse cannabinoids for a wide range of palliative care symptoms, end-of-life care in general, and adjuvant medication. Still, the gap between their beliefs and actual recommendations or prescriptions is vast.^14^ Educating the palliative care health professionals and providing accurate explanations to patients could help solve this contradiction.

The galenic form is the first point noted to distinguish therapeutic from illicit products. The drops are considered safer than inhaled formulation, contributing to lung damage and addiction. The second point concerns side effects, in particular the risk of dependence. Nevertheless, patients agree that the benefits, notably improved quality of life, outweigh the fear of side effects and becoming addicted.

A Swiss study evaluating the use of CBMs in older adults with dementia showed that relatives put the notion of dependence into perspective. For these families, their parents are already dependent on psychotropic medications, and, given their life expectancy, their comfort should advance the moral connotation of CBMs.^23^ Consumption of cannabis in cigarettes is the most widespread among recreational users and is one of the most popular among therapeutic users. However, this form of consumption should be avoided because it is associated with adverse effects such as chronic cough, bronchitis, and, above all, inhalation of toxic combustion products (carbon monoxide, tar, or ammonia, among many others).

The legal status of cannabis impacts the perception of CBMs and its use in therapy.^13^ In addition, the moral connotation inherited from older generations blocks the progress of cannabinoids and their prescription. However, with the gradual legalization of cannabis in several nations, the impression that cannabis-containing THC at a high percentage is illegal may diminish.^24^

The qualitative approach of palliative care patients' feedback that gives a rich view of their representations represents a strength of this study. Patients were randomly selected in a care setting, and half of them relatively spontaneously discussed their recreational CBM usage. Limitations include the low number of participants, all interviewed in the same setting with a possible selection bias with patients relatively favorable to the administration of CBMs, even if the patients were randomly selected.

## Conclusion

The obstacles described by the patients seem to be surmountable with specific measures at the clinical level. We suggest training the health professionals working in a palliative care setting, especially to explain the effects and side effects. In addition, randomized control trials might help define the right time for prescription, the formulation, and reliable clinical practice guidelines adapted for this population.

Furthermore, ongoing change in the legal status of cannabis in different countries, related to a shift in perception of CBMs, is encouraging. CBMs will undoubtedly play a more significant role in palliative care medicine in the years to come.

## Abbreviations Used

CBMs: cannabinoid-based medicines
FOPH: Federal Office of Public Health
HUG: University Hospital Geneva
THC: delta-9-tetrahydrocannabinol

## Appendix 1. Semistructured Interview Guide

(1) Have you used recreational/nontherapeutic cannabis in the past, or do you currently use it?
  - When did you use it? In what context?
  - Is/was it occasional or regular use?
  - What has using recreational cannabis done for you?
  - If not, how do you feel about recreational use?
(2) What do you know about medical cannabis?
  For example, (to be expanded according to the patient's answers):
  - Indication
  - Expectations
  - Side effects
  - Route of administration
  - Personal experience
  - Experience of relatives.
(3) Do you consider medical cannabis as a medicine?
(4) What type of medicine is medical cannabis for you?
  - Psychotropic
  - Antidepressant
  - Antianxiety
  - Antipain
  - Anti-inflammatory
  - Other: specify.
(5) In what form would you like to consume cannabis if you had the choice?
  - Smoked
  - Vaporized
  - In drops
  - In a tablet or capsule
  - Other: specify.
(6) Do you want to add something?
